# Respiratory Function Analysis in Patients with Chronic Pain: An Umbrella Review and Meta-Analysis of Pooled Findings

**DOI:** 10.3390/healthcare11091358

**Published:** 2023-05-08

**Authors:** Ferran Cuenca-Martínez, Núria Sempere-Rubio, Elena Muñoz-Gómez, Sara Mollà-Casanova, Enrique Carrasco-González, Francisco M. Martínez-Arnau

**Affiliations:** 1 Department of Physiotherapy, University of Valencia, 46010 Valencia, Spain; ferran.cuenca@uv.es (F.C.-M.); elena.munoz-gomez@uv.es (E.M.-G.); sara.molla@uv.es (S.M.-C.); 2 Department of Physiotherapy, Universidad Europea Miguel de Cervantes, c/del Padre Julio Chevalier nº2, 47012 Valladolid, Spain; enri.96glx@gmail.com; 3 Clínica Neuron Paseo De La Habana, c/Fray Bernardino Sahagún 9, 28036 Madrid, Spain; 4 Physiotherapy in Motion Multispeciality Research Group (PTinMOTION), Department of Physiotherapy, University of Valencia, 46010 Valencia, Spain; francisco.m.martinez@uv.es

**Keywords:** respiratory dysfunction, respiratory disorders, respiratory disturbances, chronic pain

## Abstract

Background: The main aim of this umbrella review was to assess the respiratory function in patients with chronic pain (CP), including patients with chronic neck pain (CNP), chronic low back pain (CLBP), and fibromyalgia syndrome (FMS). Methods: We searched in PubMed, PEDro, EMBASE, CINAHL, and Google Scholar (4 February 2023). The outcome measures were respiratory muscle strength (MIP/MEP) and pulmonary function (VC, MVV, FVC, FEV_1_, FEV_1_/FVC ratio, FEV_25–75_, and PEF). This review was previously registered in the international prospective register of systematic reviews, PROSPERO (CRD42023396722). The methodological quality was analyzed using AMSTAR and ROBIS scales, and the strength of the evidence was established according to the guidelines advisory committee grading criteria. To compare the outcomes reported by the studies, we calculated the standardized mean differences and the corresponding 95% confidence interval for the continuous variables. Results: Four systematic reviews with and without meta-analysis were included, from which a total of 15 primary studies were extracted. Five meta-analyses were carried out, using analyses by subgroup according to the type of CP. The meta-analyzing variables were MIP, MEP, MVV, FEV_1_, and FVC. Conclusions: Overall, patients with CP have decreased respiratory muscle strength with a moderate quality of evidence. Regarding the pulmonary function, patients with CNP showed a diminished VC, PEF, MVV, FEV_1_, and FVC, while FEV_25–75_ and the FEV_1_/FVC ratio were conserved with a limited to moderate quality of evidence. Finally, patients with FMS and CLBP only showed a decrease in MVV with a limited quality of evidence.

## 1. Introduction

The International Classification of Diseases proposes the term ‘chronic pain’ (CP) when the presence of pain is maintained beyond the normal period of tissue healing (3 months after the first occurrence of the painful episode) [1]. In fact, the International Association for the Study of Pain (known as IASP) considers that in conditions such as fibromyalgia syndrome (FMS) or chronic non-specific low back pain (CLBP), the CP may be considered as a disease in itself and not as a symptom [1]. The CP is one of the leading causes of disability and suffering worldwide [2] and is also the main reason why patients seek health care [3]. The number of people suffering from CP is very high worldwide. For example, almost 25% of Americans report having CP [4]. In Spain, it seems to be around 12%, while in the UK, it is almost 40% [5]. At the socio-economic level, CP has a very considerable impact, reaching an investment in the United States of America between USD 500 and USD 600 billion per year in medical costs [6,7]. The CP seems to be a dynamic consequence of the interaction of biological but also psychosocial factors [8]. At the pathophysiological level, CP is associated with changes in the functioning of the central nervous system, both at the medullary and supramedullary levels [9,10], including a release of proinflammatory factors such as substance P, alterations in gene expression, or a maladaptive reorganization at the cortical level, such as a substantial loss of gray brain matter or changes in some brain connections involved in the perpetuation of pain [7,11,12,13]. At the psychosocial level, patients with CP have been shown to be influenced by cognitive-evaluative variables (such as the presence of catastrophic thoughts or the role of self-efficacy expectations), emotional variables (such as the presence of pain-related fear or depressive and anxiety symptoms), and behavioral variables (such as loss of active pain management strategies or fear-related avoidance) [1]. However, one of the aspects that had not been extensively studied so far is the relationship between CP and respiratory function. Investigation of the respiratory function in patients with CP seems important because it could have a significant impact on the habitual evaluation and intervention of these patients and could lead to more thoughtful clinical decisions [14]. Even so, in recent years, there has been a growing interest in this topic [15,16,17,18]. For example, Beeckmans et al. found that the presence of lumbar pain is associated with the presence of respiratory disorders such as dyspnea, asthma, allergy, or respiratory infections [19]. Other studies, such as those conducted by López de Uralde Villanueva et al. [15] and by Kahlaee et al. [18], found that patients with chronic neck pain (CNP) presented a decrease in respiratory muscle strength and pulmonary function compared with asymptomatic individuals. Other systematic reviews (SRs) have evaluated respiratory function in patients with FMS [16] and CLBP [17].

Despite this, so far, no research has pooled all the studies evaluating respiratory function in patients with chronic pain. Therefore, this umbrella review is performed. The main aim of this umbrella review was to assess the respiratory function in patients with CP.

## 2. Materials and Methods

The present umbrella review was conducted in accordance with the Preferred Reporting Items for Overviews of Systematic Reviews including harm checklist (PRIO-harms), which consists of 27 items (56 sub-items) [20], and also according to the Preferred Reporting Items for Systematic Reviews and Meta-analysis (PRISMA) 2020 statement. The present umbrella review was previously registered in PROSPERO (CRD42023396722).

### 2.1. Inclusion Criteria

The inclusion criteria used in the study were based on patients, outcome measures, and design.

#### 2.1.1. Patients

The population were adult patients (>18 y/o) with CP (any self-reported pain in any anatomical region of at least 3 months duration). The included SRs had to explicitly state that they included patients with CP in their inclusion criteria. The comparator group should be composed of healthy subjects (healthy control group).

#### 2.1.2. Outcome Measures

The outcomes assessed were divided in respiratory muscle strength and pulmonary function.

##### Respiratory Muscle Strength

The respiratory muscle strength included maximal inspiratory pressure (MIP) and maximal expiratory pressure (MEP).

##### Pulmonary Function

The pulmonary function included vital capacity (VC), maximum voluntary ventilation (MVV), forced vital capacity (FVC), forced expiratory volume during the first second (FEV_1_), forced expiratory ratio (FEV_1_/FVC), forced expiratory flow from 25% to 75% (FEV_25–75_), and peak expiratory flow (PEF).

#### 2.1.3. Study Design

We selected SRs (with or without a meta-analysis) of observational studies (cross-sectional design or longitudinal design (cohorts or case and control studies)). There were no restrictions for any specific language, as recommended by the international criteria [21].

### 2.2. Search Strategy

We conducted the search for published scientific articles between 1950 and 4 February 2023, in the following databases: PubMed (Medline), PEDro, EMBASE, and CINAHL. An additional manual search was realized in Google Scholar. The reference sections of the included studies and original studies were screened manually, and the authors were contacted for further information if necessary. The search strategy combined Medical Subjects’ Headings (MeSH) [“respiratory physiological phenomena”], [“physiopathology”], [“respiration”], [“chronic pain”], or [“systematic reviews as topic”] and non-MeSH terms (“respiratory dysfunction”, “respiratory disturbances”, “cervical pain”, “fibromyalgia”, or “low back pain”) adding a Boolean operator (AND and/or OR) to combine them. Appendix A.1 shows the search strategy, which was adapted for each database. The search was conducted by two independent reviewers (F.C.-M. and N.S.-R.) using the same methodology. Differences that emerged during this phase were resolved by consensus. The reference sections of the original studies were screened manually, and the authors were contacted for further information if necessary.

### 2.3. Selection Criteria and Data Extraction

A total of two independent researchers conducted a screening (F.C.-M. and N.S.-R.) assessing the relevance of the SRs regarding the studies’ questions and aims. The first screening was based on each study’s title information and abstract. In the second phase of the screening, the complete text was assessed if the SRs met all of the inclusion criteria. Differences between the reviewers were resolved by a consensus process mediated by a third researcher (F.M.M.-A.).

### 2.4. Methodological Quality Assessment

The two independent researchers (F.C.-M. and N.S.-R.) assessed the methodological quality of the primary studies, evaluating each of the selected studies based on the Modified Quality Assessment Scale (AMSTAR) developed by Barton et al. [22], a scale shown to be a valid and reliable tool for assessing the methodological quality of SRs. With a total of 13 items, each worth two points (“yes” = two points; “in part” = one point; “no” = zero points), the maximum possible score is 26 points. A high-quality cutoff of 20 or more points was provided by the developers. In addition, we calculated the kappa coefficient (κ) to assess reliability prior to any consensus [23].

### 2.5. Risk of Bias Assessment

We assessed the risk of bias with the risk of bias in SR tool (ROBIS) [24], which consists of three phases: (1) relevance assessment (optional); (2) identification of concerns with the review process through four domains related to studying eligibility criteria, identification and selection of studies, data collection and study appraisal, and synthesis and findings; and (3) judgment on the risk of bias. In addition, we calculated the kappa coefficient (κ) and percentage (%) agreement scores to assess the reliability prior to any consensus.

### 2.6. Grading of Evidence

The physical activity guidelines advisory committee grading criteria (PAGAC) were used to assess the grading of evidence, evaluating the following criteria: (1) applicability (2) generalizability to the population, (3) risk of bias, (4) consistency of results, and (5) magnitude of the effect [25].

### 2.7. Data Synthesis and Statistical Analysis

For the statistical aggregation of the pooled findings (primary studies), all analyses were performed using Meta XL, v. 5.3 (EpiGear International, Queensland, Australia) [26]. We calculated the standardized mean differences (SMDs) with the 95% of confidence interval (CI) for the dependent variables. The statistical significance was examined as Hedges’ *g*. The estimated SMDs were interpreted as described by Hopkins et al. [27]; that is, we considered that an SMD of 4.0 represented an extremely large clinical effect, 2.0–4.0 represented a very large effect, 1.2–2.0 represented a large effect, 0.6–1.2 represented a moderate effect, 0.2–0.6 represented a small effect, and 0.0–0.2 represented a trivial effect. To detect publication bias, we performed a visual evaluation of the DOI plot [28], seeking asymmetry, as well as a quantitative determination of the Luis Furuya-Kanamori (LFK) index, an index known to be more sensitive than the Egger test in detecting publication bias in meta-analyses with low numbers of studies [29]. LFK indexes less than ±1 represent no asymmetry, those greater than ±1 but less than ±2 represent minor asymmetry, and those greater than ±2 indicate major asymmetry [29]. We estimated the degree of heterogeneity among the study outcomes by employing Cochran’s Q statistic test (with *p* < 0.1 considered significant) and the inconsistency index (I^2^) [30], with I^2^ > 25% indicating low heterogeneity, I^2^ > 50% indicating medium heterogeneity, and I^2^ > 75% indicating large heterogeneity [31]. The I^2^ index complements the Q test, although the I^2^ index has comparable issues regarding power when applied to a small number of studies [31]. Therefore, studies were considered heterogeneous when they fulfilled one or both of the following conditions: (1) the Q test was significant (*p* < 0.1), and (2) I^2^ was >75%. To obtain a pooled estimate of the effect in the meta-analysis of the heterogeneous studies, we assumed a random-effects model, as described by DerSimonian and Laird [32].

## 3. Results

### 3.1. Study Selection

**Figure 1:**
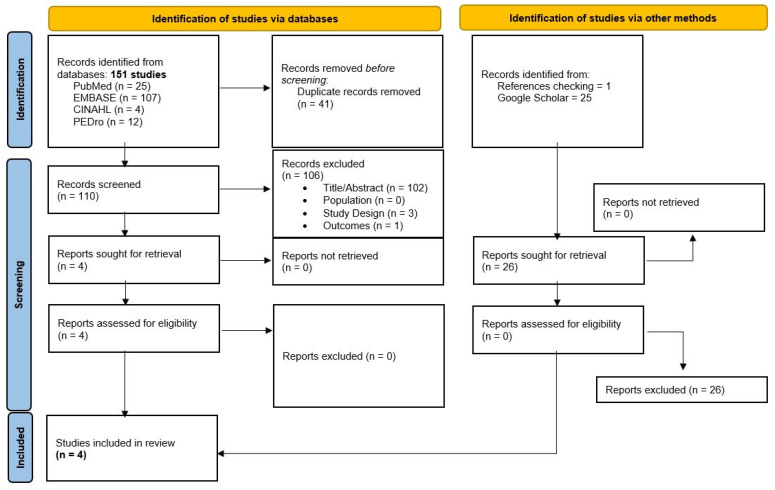
PRISMA flow chart of studies selection.

The initial search revealed 151 records. Through the title and abstract screening and the full-text assessment, four SRs were eligible according to our criteria [15,16,17,18]. The study screening strategy is shown in the form of a flow chart (Figure 1). From the four SRs that were included, the primary studies were extracted in order to perform the grouped meta-analyses in the present umbrella review. From the SRs conducted by López de Uralde Villanueva et al. [15] and Kahlaee et al. [18] in patients with CNP, the following primary studies were extracted: [33,34,35,36,37,38,39,40]. The following primary studies were extracted from the SRs conducted by Ortiz-Rubio et al. [16] in patients with FMS: [41,42,43,44,45]. Finally, only two primary studies were extracted from the study by Abdollahzadeh and Abbasi [17] in patients with CLBP [46,47], because they were the only ones that adhered to the aim of this umbrella review.

### 3.2. Characteristics of the Included Systematic Reviews

Table 1 lists the characteristics of the SRs included (study design, original studies included, demographic characteristics, interventions, variables, and results).

### 3.3. Results of AMSTAR and ROBIS

The scores ranged from 15 to 25 points out of a possible 26, with a mean score of 20 points. Two (50%) studies scored above 20 points and were considered high quality (Table 2). The items with the highest scores were those related to “explicitly described to allow replication” or “quality assessment explicitly described to allow replication”. The lowest scoring item was “conclusions address levels of evidence for each comparison”. The inter-rater reliability of the methodological quality assessment was high (κ = 0.93).

**Figure 2:**
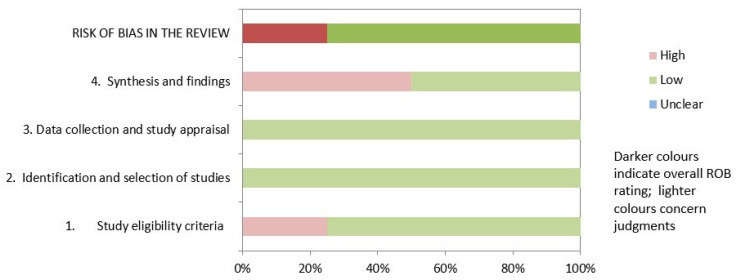
Graphical representation for ROBIS results.

Table 3 and Figure 2 show the results of the risk of bias assessment using ROBIS. Of the studies, 75% showed low risk of bias. The domain related to the “study eligibility criteria” had the highest risk of bias. In contrast, the domain related to the “identification and selection of studies” and “data collection and study appraisal” had the lowest risk of bias. The inter-rater reliability of the methodological quality assessment was high (κ = 0.77).

### 3.4. Grading of Evidence Results (PAGAC)

Table 4 shows the quality of evidence. The quality of evidence found for MIP, MEP, FVC, and FEV_1_ outcome measures was moderate, while for the VC, MVV, FEV_1_/FVC ratio, FEV_25–75_, and PEF, the variables were limited.

### 3.5. Qualitative Synthesis

All pulmonary function variables analyzed in the qualitative synthesis section were extracted from the study by López de Uralde Villanueva et al. [15] and were therefore assessed only in the CNP population. These variables (VC, FEV_1_/FVC ratio, FEV_25–75_, and PEF) could not be included in the meta-analyses of the pooled findings because they were not analyzed in the remaining clinical populations (FMS and CLBP).

#### 3.5.1. Vital Capacity

The study conducted by López de Uralde Villanueva et al. [15] found that patients with CNP showed a diminished VC with a small clinical effect and without evidence of significant heterogeneity (n = 4, SMD = −0.31 (−0.56 to −0.06), Q = 1.19, *p* = 0.76, I^2^ = 0%).

#### 3.5.2. Forced Expiratory Ratio

The study conducted by López de Uralde Villanueva et al. [15] found no significant differences regarding the FEV_1_/FVC ratio in patients with CNP with evidence of significant heterogeneity (n = 7, SMD = 0.10 (−0.57 to 0.77), Q = 93.86, *p* < 0.01, I^2^ = 93.6%).

#### 3.5.3. Forced Expiratory Flow from 25% to 75%

The study conducted by López de Uralde Villanueva et al. [15] found no significant differences regarding FEV_25–75_ in patients with CNP with no evidence of significant heterogeneity (n = 4, SMD = −0.18 (−0.45 to 0.10), Q = 5.44, *p* = 0.14, I^2^ = 44.9%).

#### 3.5.4. Peak Expiratory Flow

The study conducted by López de Uralde Villanueva et al. [15] found that patients with CNP showed a diminished PEF with a small clinical effect and with evidence of significant heterogeneity (n = 6, SMD = −0.58 (−1.03 to −0.12), Q = 29.53, *p* < 0.01, I^2^ = 83.1%).

### 3.6. Meta-Analysis Results

#### 3.6.1. Maximal Inspiratory Pressure

**Figure 3:**
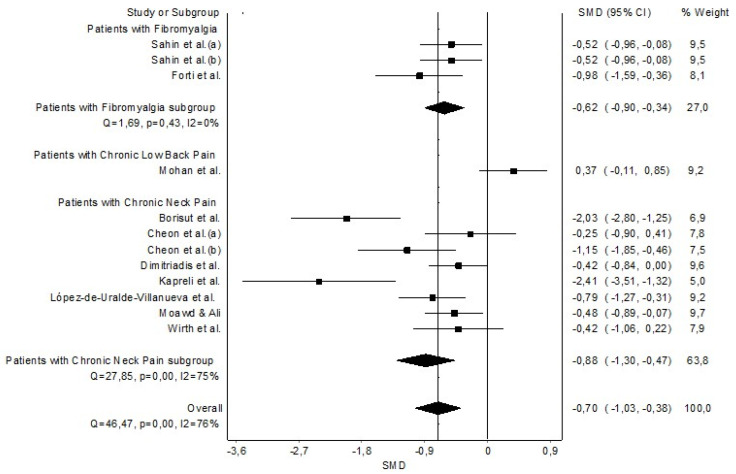
Synthesis forest plot of maximal inspiratory pressure. The small boxes with the squares represent the point estimate of the effect size and sample size. The lines on either side of the box represent a 95% confidence interval (CI). Sahin et al. (a) [43]; Sahin et al. (b) [44]; Forti et al. [42]; Mohan et al. [47]; Borisut et al. [34]; Cheon et al. (a)(b) [33], Dimitriadis et al. [35]; Kapreli et al. [36]; López-de-Uralde-Villanueva et al. [37]; Moawd & Ali [38] and Wirth et al. [39].

The grouped meta-analysis found statistically significant evidence with a moderate clinical effect that MIP was decreased in patients with CP (n = 11, SMD = −0.70 (−1.03 to −0.38)) with evidence of significant heterogeneity (Q = 46.47, *p* < 0.001, I^2^ = 76%) (Figure 3). The DOI and funnel plot showed certain asymmetry, and the LFK index presented minor asymmetry (LFK = −1.76), indicating a low to moderate risk of publication bias (Appendix A.2). Regarding the sensitivity analysis, significant strong results were obtained since the analysis suggested that no individual studies affected the pooled SMDs (Appendix A.3).

#### 3.6.2. Maximal Expiratory Pressure

**Figure 4:**
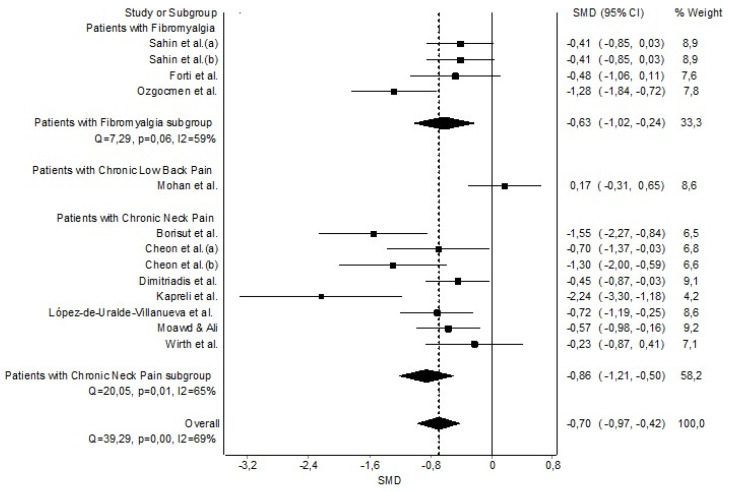
Synthesis forest plot of maximal expiratory pressure. The small boxes with the squares represent the point estimate of the effect size and sample size. The lines on either side of the box represent a 95% confidence interval (CI). Sahin et al. (a) [43]; Sahin et al. (b) [44]; Forti et al. [42]; Ozgocmen et al. [45]; Mohan et al. [47]; Borisut et al. [34]; Cheon et al. (a)(b) [33], Dimitriadis et al. [35]; Kapreli et al. [36]; López-de-Uralde-Villanueva et al. [37]; Moawd & Ali [38] and Wirth et al. [39].

The grouped meta-analysis found statistically significant evidence with a moderate clinical effect that MEP was decreased in patients with CP (n = 12, SMD = −0.70 (−0.97 to −0.42)) with evidence of significant heterogeneity (Q = 39.29, *p* < 0.001, I^2^ = 69%) (Figure 4). The shape of the funnel and DOI plot presented asymmetry, and the LFK index showed major asymmetry (LFK = −2.03), indicating a high risk of publication bias (Appendix A.4). Regarding the sensitivity analysis, significant strong results were obtained since the analysis suggested that no individual studies affected the pooled SMDs (Appendix A.5).

#### 3.6.3. Maximum Voluntary Ventilation

**Figure 5:**
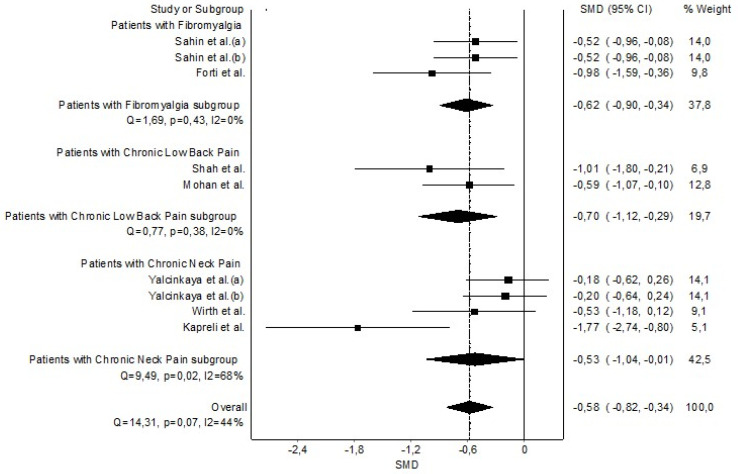
Synthesis forest plot of maximum voluntary ventilation. The small boxes with the squares represent the point estimate of the effect size and sample size. The lines on either side of the box represent a 95% confidence interval (CI). Sahin et al. (a) [43]; Sahin et al. (b) [44]; Forti et al. [42]; Shah et al. [46]; Mohan et al. [47]; Yalcinkaya et al. (a)(b) [40]; Wirth et al. [39]; and Kapreli et al. [36].

The grouped meta-analysis found statistically significant evidence with a small clinical effect that MVV was decreased in patients with CP (n = 8, SMD = −0.58 (−0.82 to −0.34)) with no evidence of significant heterogeneity (Q = 14.31, *p* = 0.07, I^2^ = 44%) (Figure 5). The shape of the funnel and DOI plot presented asymmetry, and the LFK index showed major asymmetry (LFK = −2.06), indicating a high risk of publication bias (Appendix A.6). The influence of each individual study was assessed with a sensitivity exclusion analysis. Statistically weak results were obtained, given the analysis suggested that five studies significantly affected the pooled SMDs (Appendix A.7).

#### 3.6.4. Forced Vital Capacity

**Figure 6:**
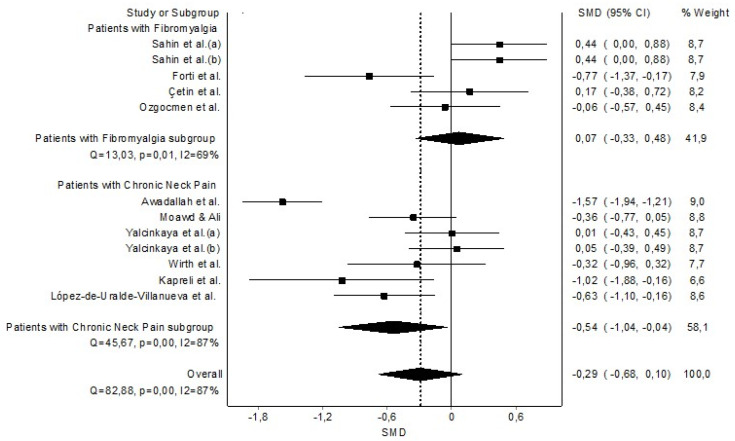
Synthesis forest plot of forced vital capacity variable. The small boxes with the squares represent the point estimate of the effect size and sample size. The lines on either side of the box represent a 95% confidence interval (CI). Sahin et al. (a) [43]; Sahin et al. (b) [44]; Forti et al. [42]; Çetin et al. [41]; Ozgocmen et al. [45]; Moawd & Ali [38]; Yalcinkaya et al. (a)(b) [40]; Wirth et al. [39]; Kapreli et al. [36]; and López-de-Uralde-Villanueva et al. [37].

The grouped meta-analysis found no statistically significant evidence that FVC was decreased in patients with CP (n = 11, SMD = −0.29 (−0.68 to 0.10)) with evidence of significant heterogeneity (Q = 82.88, *p* < 0.001, I^2^ = 87%) (Figure 6). In addition, the analysis by subgroups found that FVC was decreased only in patients with CNP with a moderate clinical effect (n = 6, SMD = −0.54 (−1.04 to −0.04)) but not in patients with FMS (n = 5, SMD = 0.07 (−0.33 to 0.48)) (Figure 6). The DOI and funnel plot did not present asymmetry, and the LFK index showed no asymmetry (LFK = −0.99). Therefore, we found a very low risk of publication bias (Appendix A.8).

#### 3.6.5. Forced Expiratory Volume during the First Second

**Figure 7:**
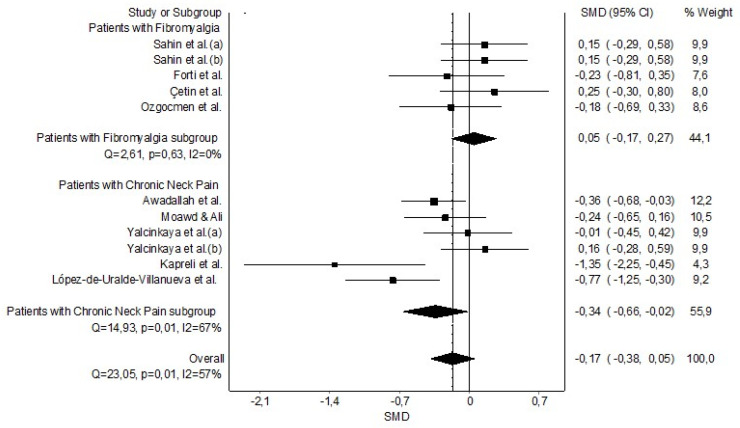
Synthesis forest plot of forced expiratory volume during the first second variable. The small boxes with the squares represent the point estimate of the effect size and sample size. The lines on either side of the box represent a 95% confidence interval (CI). Sahin et al. (a) [43]; Sahin et al. (b) [44]; Forti et al. [42]; Çetin et al. [41]; Ozgocmen et al. [45]; Moawd & Ali [38]; Yalcinkaya et al. (a)(b) [40]; Wirth et al. [39]; Kapreli et al. [36]; and López-de-Uralde-Villanueva et al. [37].

The grouped meta-analysis found no statistically significant evidence that FEV_1_ was decreased in patients with CP (n = 10, SMD = −0.17 (−0.38 to 0.005)) with evidence of significant heterogeneity (Q = 23.05, *p* = 0.01, I^2^ = 77%) (Figure 7). In addition, the analysis by subgroups found that FEV_1_ was decreased only in patients with CNP with a moderate clinical effect (n = 5, SMD = −0.34 (−0.66 to −0.02)) but not in patients with FMS (n = 5, SMD = 0.05 (−0.17 to 0.27)) (Figure 7). The DOI and funnel plot did not present asymmetry, and the LFK index showed no asymmetry (LFK = −0.39). Therefore, we found a very low risk of publication bias (Appendix A.9).

## 4. Discussion

The main aim of this umbrella review was to assess the respiratory function in patients with CP. The results showed that patients with CP have decreased respiratory muscle strength, both inspiratory and expiratory, as well as some pulmonary function parameters such as VC, PEF, or MVV. Other lung function parameters are preserved, such as FEV_25–75_ or the FEV_1_/FVC ratio. However, we performed subgroup analyses with the aim of jointly but also separately assessing some respiratory function variables. Our subgroup analysis found that FEV_1_ and FVC are not decreased in a grouped manner in patients with CP but are diminished in patients with CNP specifically.

Patients with CP present somatosensory and also motor changes compared with healthy subjects such as proprioceptive alterations, abnormalities in motor control, or loss of active range of motion [48,49,50]. These sensorimotor changes could affect respiratory function and may justify the findings found in this umbrella review. For example, patients with CNP have shown an inefficient pattern of muscle activity of the superficial cervical flexor muscles due to cranio-cervical motor control disorder characterized by hyperactivity [51,52]. This could lead to fatigue of these muscles, which are involved in breathing both at tidal volume and in forced inspiratory maneuvers, for their role as accessory muscles of inspiration. An alteration at this level could affect both inspiratory force and lung filling capacity, including the MVV which is an endurance test of the respiratory musculature. In addition to this, decreasing the lung filling capacity could imply a lower lung expansion that could lead to a decrease in lung volumes, thus justifying some of the findings found in the study, such as the decrease in VC or FVC. All these arguments were used by López de Uralde Villanueva et al. [15] to justify their findings.

Regarding the patients with CLBP, it seems that the diaphragm is a key point to be analyzed. Ruhe et al. [53] showed a close relationship between loss of postural control and the presence of low back pain. The diaphragm muscle, which actively participates in inspiration and, therefore, in pulmonary filling, seems to play a key role in postural control [54]. Janssens et al. [55] found that people with CLBP showed an increased susceptibility to diaphragm fatigue compared with asymptomatic subjects. Kolár et al. [56] found that the diaphragm has a lower excursion during inspiration in patients with CLBP, as well as increased fatigability. Increased fatigue of the diaphragm could lead to decreased respiratory function in general and could explain the findings found in the MVV test. Even so, the studies that included patients with CLBP were very few, making it very difficult to conclude consistently for this population.

Finally, regarding the patients with FMS, the SR conducted by Ortiz-Rubio et al. [16] offers some interesting justifications to be taken into account. Patients with FMS present a number of clinical problems that could lead to respiratory alterations [57]. For example, patients with FMS have shown less chest box expansion compared with subjects without pathology [16]. This could be related to a decrease in inspiratory force. In fact, some studies have found a relationship between reduced thoracic expansion and a decrease in respiratory musculature strength [43,44]. A loss of respiratory musculature strength could lead to a reduced ability to perform the MVV test and also a decrease in lung filling capacity and could affect some ventilatory function parameters [43,44,45,58]. Moreover, modifications in chest box expansion could also lead to respiratory alterations by influencing the length–tension curve of the abdominal muscles, diaphragm, or external intercostals [16]. Finally, FMS is a clinical entity that presents with generalized pain, as well as being closely related to some comorbidities such as cervical pain or myofascial pain syndromes, which could directly influence a loss of respiratory function [16].

### 4.1. Clinical Implications

At the clinical level, it should be considered that patients with CP might have decreased respiratory muscle strength, as well as alteration of different pulmonary function parameters, probably due to the former. According to this umbrella review, as well as the SRs reviewed for this study, future studies should include a respiratory muscle training program to improve their values, as well as to secondarily improve the pulmonary function. We believe that future clinical studies in the field of CP should perform specific therapeutic exercise programming of the respiratory musculature, as well as generally including aerobic and strength exercise for this aim along with other clinical interventions including manual therapy, therapeutic education, pharmacological therapy, or psychological intervention with the aim of addressing the patient with CP in a comprehensive manner. Finally, at the research level, more studies should be conducted to assess respiratory function in more conditions with chronic pain and then, if altered, include them in the treatment targets.

### 4.2. Limitations

This article has some limitations that should be taken into consideration. First, the patients evaluated were different. We have evaluated musculoskeletal and rheumatologic patients, causing some heterogeneity that should be considered as an important limitation. Second, some pulmonary function variables were evaluated only in one clinical population (CNP), so that they could not be pooled in a meta-analysis. Third, very few primary studies were found in persistent low back pain, so that more research could probably change the result obtained in this study. Fourth, we grouped some pulmonary function variables that were expressed in different units (total value in liters or percentage with respect to the theoretical value), so we had to use a standardization of the mean differences to be able to compare the results. Finally, other analyses by subgroups would have been interesting, for example, by gender or pain intensity, but due to the mix of data in the studies, it was not possible to perform them.

## 5. Conclusions

Overall, patients with CP have decreased respiratory muscle strength with a moderate quality of evidence. Regarding the pulmonary function, patients with CNP showed diminished VC, PEF, MVV, FEV_1_, and FVC, while the FEV_25–75_ and FEV_1_/FVC ratio variables were conserved with a limited to moderate quality of evidence. Finally, patients with FMS and CLBP only showed a decrease in MVV with a limited quality of evidence.

## Figures and Tables

**Table 1:** Characteristics of the reviews included in the umbrella review.

Study	Studies k (n) Types	Meta-Analysis (k)	Population	Variables	Outcomes (Test)	Author’s Conclusions
López-de-Uralde-Villanueva et al. [15]	11 OSs (929)	Yes (10)	-Patients with CNP -HC	-Pulmonary function (VC, MVV, FVC, FEV_1_, FEV_1_/FVC, FEV_25–75_, and PEF) -Respiratory muscle strength (MIP and MEP)	-Pulmonary function (spirometry test) -Respiratory muscle strength (portable mouth pressure meter)	Patients with CNP have reduced respiratory muscle strength and pulmonary function compared with HC.
Ortiz-Rubio et al. [16]	Seven OSs (504)	Yes (6)	-Patients with FMS -HC	-Pulmonary function (MVV, FVC, and FEV_1_) -Respiratory muscle strength (MIP and MEP)	-Pulmonary function (spirometry test) -Respiratory muscle strength (digital mouth pressure meter)	Patients with FMS experience respiratory disturbances in comparison with HC.
Abdollahzadeh and Abbasi [17]	Seven OSs (N/R)	No	-Patients with CLBP -HC	-Pulmonary function (MVV) -Respiratory muscle strength (MIP and MEP)	-Pulmonary function (spirometry test) -Respiratory muscle strength (portable mouth pressure meter)	A sub-optimal respiratory function was found in patients with CLBP.
Hossein-Kahlaee et al. [18]	Nine OSs (585)	No	-Patients with CNP -HC	-Pulmonary function (VC, MVV, FVC, FEV_1_, FEV_1_/FVC, and PEF) -Respiratory muscle strength (MIP and MEP)	-Pulmonary function (spirometry test) -Respiratory muscle strength (portable mouth pressure meter)	Patients with CNP have reduced respiratory muscle strength and pulmonary function compared with HC.

Abbreviations: OS: observational study; CNP: chronic neck pain; HC: healthy controls; VC: vital capacity; MVV: maximum voluntary ventilation; FVC: forced vital capacity; FEV_1_: forced expiratory volume during the first second; FEV_1_/FVC: forced expiratory ratio; FEV_25–75_: forced expiratory flow from 25% to 75%; PEF: peak expiratory flow; MIP: maximal inspiratory pressure; MEP: maximal expiratory pressure; FMS: fibromyalgia syndrome; CLBP: chronic low back pain; N/R: not reported. Note: The primary systematic reviews could have included more variables that, not being of interest to this study, were not included in Table 1.

**Table 2:** Quality assessment scores (AMSTAR).

Study	1	2	3	4	5	6	7	8	9	10	11	12	13	Score
López-de-Uralde-Villanueva [15]	2	2	2	2	2	2	2	2	2	2	2	2	1	25
Ortiz-Rubio et al. [16]	2	2	2	2	1	2	0	2	2	2	2	2	1	22
Abdollahzadeh and Abbasi [17]	2	1	0	2	0	2	2	2	2	0	0	2	0	15
Hossein-Kahlaee et al. [18]	2	2	2	2	0	2	2	2	2	0	0	2	0	18

Notes: maximum score = 26. High-quality cutoff ≥ 20.

**Table 3:** Risk of bias assessment in systematic reviews through the ROBIS scale.

Study	Phase 2				Phase 3
	Study Eligibility Criteria	Identification and Selection of Studies	Data Collection and Study Appraisal	Synthesis and Findings	Risk of Bias in the Review
López-de-Uralde-Villanueva [15]	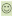	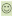	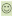	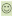	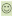
Ortiz-Rubio et al. [16]	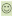	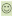	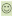	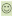	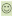
Abdollahzadeh and Abbasi [17]	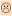	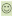	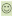	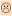	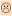
Hossein-Kahlaee et al. [18]	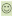	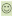	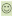	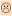	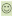

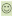
: low risk. 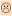: high risk.

**Table 4:** Summary of findings and quality of evidence (PAGAC).

2018 Physical Activity Guidelines Advisory Committee Grading Criteria						Grade
Systematic Review Research Questions	Applicability	Generalizability	Risk of bias or study limitations	Quantity and consistency	Magnitude and precision of effect	
MIP	Strong	Limited	Moderate	Moderate	−0.70 (−1.03 to −0.38)	Moderate
MEP	Strong	Limited	Moderate	Moderate	−0.70 (−0.97 to −0.42)	Moderate
VC	Strong	Limited	Moderate	Limited	Not assignable	Limited
MVV	Strong	Limited	Moderate	Limited	−0.58 (−0.82 to −0.34)	Limited
FVC	Strong	Limited	Moderate	Moderate	−0.29 (−0.68 to 0.10)	Moderate
FEV_1_	Strong	Limited	Moderate	Moderate	−0.17 (−0.38 to 0.005)	Moderate
FEV_1_/FVC	Strong	Limited	Moderate	Limited	Not assignable	Limited
FEV_25–75_	Strong	Limited	Moderate	Limited	Not assignable	Limited
PEF	Strong	Limited	Moderate	Limited	Not assignable	Limited

Abbreviations: MIP: maximal inspiratory pressure; MEP: maximal expiratory pressure; VC: vital capacity; MVV: maximum voluntary ventilation; FVC: forced vital capacity; FEV_1_: forced expiratory volume during the first second; FEV_1_/FVC: forced expiratory ratio; FEV_25–75_: forced expiratory flow from 25% to 75%; and PEF: peak expiratory flow.

## Data Availability

Contact the corresponding author (N.S.-R.).

## Abbreviations

Vital capacity (VC); maximum voluntary ventilation (MVV); forced vital capacity (FVC); forced expiratory volume during the first second (FEV_1_); forced expiratory ratio (FEV_1_/FVC); forced expiratory flow from 25% to 75% (FEV_25–75_); peak expiratory flow (PEF); maximal inspiratory pressure (MIP); maximal expiratory pressure (MEP); chronic pain (CP); chronic neck pain (CNP); chronic low back pain (CLBP); and fibromyalgia syndrome (FMS).

## Appendix A

### Appendix A.1. Database Search Strategies

-PubMed (Medline)

1-(“respiratory physiological phenomena”[MeSH Terms] OR (“Respiratory”[All Fields] AND “physiological”[All Fields] AND “phenomena”[All Fields]) OR “respiratory physiological phenomena”[All Fields] OR (“Respiratory”[All Fields] AND “Function”[All Fields]) OR “respiratory function”[All Fields] OR “respiration”[MeSH Terms] OR “respiration”[All Fields] OR (“Respiratory”[All Fields] AND “Function”[All Fields]) OR ((“eur med j respir”[Journal] OR “Respiratory”[All Fields]) AND (“dysfunctional”[All Fields] OR “dysfunctionals”[All Fields] OR “dysfunctioning”[All Fields] OR “dysfunctions”[All Fields] OR “physiopathology”[MeSH Subheading] OR “physiopathology”[All Fields] OR “dysfunction”[All Fields])) OR (“j reticuloendothel soc”[Journal] OR “res”[All Fields]) OR ((“eur med j respir”[Journal] OR “Respiratory”[All Fields]) AND (“disturb”[All Fields] OR “disturbance”[All Fields] OR “disturbances”[All Fields] OR “disturbancies”[All Fields] OR “disturbed”[All Fields] OR “disturbing”[All Fields] OR “disturbs”[All Fields])) OR ((“eur med j respir”[Journal] OR “Respiratory”[All Fields]) AND (“disease”[MeSH Terms] OR “disease”[All Fields] OR “disorder”[All Fields] OR “disorders”[All Fields] OR “disorder s”[All Fields] OR “disordes”[All Fields]))) AND ((“orofacial pain”[Title/Abstract] OR “chronic pain”[Title/Abstract] OR “temporomandibular disorders”[Title/Abstract] OR “neck pain”[Title/Abstract] OR “low back pain”[Title/Abstract] OR “fibromyalgia”[Title/Abstract] OR “persistent pain”[Title/Abstract] OR “ache”[Title/Abstract] OR (“migrain”[All Fields] OR “migraine disorders”[MeSH Terms] OR (“migraine”[All Fields] AND “disorders”[All Fields]) OR “migraine disorders”[All Fields] OR “migraine”[All Fields] OR “migraines”[All Fields] OR “migraine s”[All Fields] OR “migraineous”[All Fields] OR “migrainers”[All Fields] OR “migrainous”[All Fields] OR (“tension type headache”[MeSH Terms] OR (“tension type”[All Fields] AND “headache”[All Fields]) OR “tension type headache”[All Fields] OR (“tension”[All Fields] AND “type”[All Fields] AND “headache”[All Fields]) OR “tension type headache”[All Fields]) OR (“headache”[MeSH Terms] OR “headache”[All Fields] OR “headaches”[All Fields] OR “headache s”[All Fields]))) AND (“respiratory dysfunction”[Title/Abstract] OR “pulmonary function”[Title/Abstract]) AND “systematic review”[Title/Abstract]) AND “systematic review”[Title/Abstract]

2-(“orofacial pain”[Title/Abstract] OR “chronic pain”[Title/Abstract] OR “temporomandibular disorders”[Title/Abstract] OR “neck pain”[Title/Abstract] OR “low back pain”[Title/Abstract] OR “fibromyalgia”[Title/Abstract] OR “persistent pain”[Title/Abstract] OR “ache”[Title/Abstract] OR (“migrain”[All Fields] OR “migraine disorders”[MeSH Terms] OR (“migraine”[All Fields] AND “disorders”[All Fields]) OR “migraine disorders”[All Fields] OR “migraine”[All Fields] OR “migraines”[All Fields] OR “migraine s”[All Fields] OR “migraineous”[All Fields] OR “migrainers”[All Fields] OR “migrainous”[All Fields] OR (“tension type headache”[MeSH Terms] OR (“tension type”[All Fields] AND “headache”[All Fields]) OR “tension type headache”[All Fields] OR (“tension”[All Fields] AND “type”[All Fields] AND “headache”[All Fields]) OR “tension type headache”[All Fields]) OR (“headache”[MeSH Terms] OR “headache”[All Fields] OR “headaches”[All Fields] OR “headache s”[All Fields]))) AND (“respiratory dysfunction”[Title/Abstract] OR “pulmonary function”[Title/Abstract]) AND “systematic review”[Title/Abstract]

3-(“respiratory function”[Title/Abstract] OR “respiratory dysfunction”[Title/Abstract] OR “respiratory disturbances”[Title/Abstract] OR “respiratory disorders”[Title/Abstract] OR “pulmonary function”[Title/Abstract] OR “pulmonary dysfunction”[Title/Abstract] OR “pulmonary disturbances”[Title/Abstract] OR “pulmonary disorders”[Title/Abstract]) AND (“chronic pain”[MeSH Terms] OR (“Chronic”[All Fields] AND “pain”[All Fields]) OR “chronic pain”[All Fields] OR “chronic pain”[Title/Abstract] OR “neck pain”[Title/Abstract] OR “cervical pain”[Title/Abstract] OR “back pain”[Title/Abstract] OR “low back pain”[Title/Abstract] OR “chronic low back pain”[Title/Abstract] OR “fibromyalgia”[Title/Abstract]) AND (“systematic review”[Publication Type] OR “systematic reviews as topic”[MeSH Terms] OR “systematic review”[All Fields] OR “systematic review”[Title/Abstract] OR “meta-analysis”[Title/Abstract])

-EMBASE

(‘chronic pain’/exp OR ‘chronic intractable pain’ OR ‘chronic pain’ OR ‘pain, chronic’ OR ‘chronic neck pain’/exp OR ‘neck pain’/exp OR ‘cervicalgia’ OR ‘neck pain’ OR ‘pain, neck’ OR ‘low back pain’/exp OR ‘acute low back pain’ OR ‘back pain, low’ OR ‘chronic low back pain’ OR ‘loin pain’ OR ‘low back pain’ OR ‘low backache’ OR ‘low backpain’ OR ‘lowback pain’ OR ‘lower back pain’ OR ‘lumbago’ OR ‘lumbal pain’ OR ‘lumbal syndrome’ OR ‘lumbalgesia’ OR ‘lumbalgia’ OR ‘lumbar pain’ OR ‘lumbar spine syndrome’ OR ‘lumbodynia’ OR ‘lumbosacral pain’ OR ‘lumbosacral root syndrome’ OR ‘lumbosacroiliac strain’ OR ‘pain, low back’ OR ‘pain, lumbosacral’ OR ‘strain, lumbosacroiliac’ OR ‘fibromyalgia’/exp OR ‘fibromyalgia’ OR ‘fibrositic nodule’ OR ‘fibrositis’ OR ‘fibrositis syndrome’ OR ‘myalgia, fibro’ OR ‘shoulder pain’/exp OR ‘pain, shoulder’ OR ‘painful shoulder syndrome’ OR ‘shoulder pain’) AND (‘lung function test’/exp OR ‘function test, lung’ OR ‘function test, pulmonary’ OR ‘lung function test’ OR ‘pulmonary function test’ OR ‘respiratory function test’ OR ‘respiratory function tests’ OR ‘respiratory test’ OR ‘ventilation test’ OR ‘forced expiratory volume’/exp OR ‘fev’ OR ‘expiration index, forced’ OR ‘expiration volume, forced’ OR ‘fev 1’ OR ‘forced expiration index’ OR ‘forced expiration test’ OR ‘forced expiration volume’ OR ‘forced expiration, maximum’ OR ‘forced expiratory index’ OR ‘forced expiratory one second volume’ OR ‘forced expiratory volume’ OR ‘lung forced expiratory volume’ OR ‘lung maximal expiration volume’ OR ‘lung maximum expiratory volume’ OR ‘lung maximum expired volume’ OR ‘lung vital capacity’ OR ‘maximal expiration’ OR ‘maximal expiratory volume’ OR ‘maximal forced expiration’ OR ‘maximal inspiratory volume’ OR ‘maximal ventilation’ OR ‘maximum expiratory lung volume’ OR ‘maximum expiratory volume’ OR ‘maximum forced expiration’ OR ‘maximum lung capacity’ OR ‘one second forced expiratory volume’ OR ‘spirometry’/exp OR ‘breath measurement’ OR ‘spirometry’ OR ‘respiratory muscle strength’/exp OR ‘lung function’/exp OR ‘function, lung’ OR ‘lung function’ OR ‘pulmonary function’ OR ‘regional lung function’) AND (‘systematic review’/exp OR ‘review, systematic’ OR ‘systematic review’ OR ‘meta analysis’/exp OR ‘analysis, meta’ OR ‘meta analysis’ OR ‘meta-analysis’ OR ‘metaanalysis’)

-PEDro

1. Abstract & Title: Chronic Pain AND Pulmonary Dysfunction. Method: systematic review (1 articles retrieved)

2. Abstract & Title: Chronic Pain AND Respiratory Dysfunction. Method: systematic review (3 articles retrieved)

3. Abstract & Title: Chronic Pain AND Pulmonary Function. Method: systematic review (4 articles retrieved)

4. Abstract & Title: Chronic Pain AND Respiratory Function. Method: systematic review (4 articles retrieved)

-CINAHL (EBSCO)

(chronic pain or persistent pain or long term pain or long-term pain) AND (pulmonary function or cardiopulmonary function or lung function or respiratory function) AND (systematic review or meta-analysis)

(neck pain or cervical pain or neck disability or neck disorder or non specific neck pain or lumbar pain or lumbopelvic pain or fibromyalgia) AND (pulmonary function or cardiopulmonary function or lung function or respiratory function) AND (systematic review or meta-analysis)
